# Techniques and Methods for Testing the Postural Function in Healthy and Pathological Subjects

**DOI:** 10.1155/2015/891390

**Published:** 2015-11-12

**Authors:** Thierry Paillard, Frédéric Noé

**Affiliations:** Physical Activity, Performance and Health Laboratory, Department of STAPS, University of Pau and Pays de l'Adour, ZA Bastillac Sud, 65000 Tarbes, France

## Abstract

The different techniques and methods employed as well as the different quantitative and qualitative variables measured in order to objectify postural control are often chosen without taking into account the population studied, the objective of the postural test, and the environmental conditions. For these reasons, the aim of this review was to present and justify the different testing techniques and methods with their different quantitative and qualitative variables to make it possible to precisely evaluate each sensory, central, and motor component of the postural function according to the experiment protocol under consideration. The main practical and technological methods and techniques used in evaluating postural control were explained and justified according to the experimental protocol defined. The main postural conditions (postural stance, visual condition, balance condition, and test duration) were also analyzed. Moreover, the mechanistic exploration of the postural function often requires implementing disturbing postural conditions by using motor disturbance (mechanical disturbance), sensory stimulation (sensory manipulation), and/or cognitive disturbance (cognitive task associated with maintaining postural balance) protocols. Each type of disturbance was tackled in order to facilitate understanding of subtle postural control mechanisms and the means to explore them.

## 1. Introduction

The ability to maintain body balance depends on complex organization which is developed with sensory inputs and is based on body geometry (segmental organization), kinetics (ground force reaction), and body orientation and vertical perception (subjective verticality) cues [1]. Pathologies disturbing sensory output, force/movement control, and spatial orientation logically affect postural control [1]. Overall, all pathologies which alter organs specifically involved in the control of posture and movement degrade postural control. For instance, Alzheimer's and Parkinson's diseases, cerebellar and vestibular syndromes, low-vision and ankle sprains, which, respectively, affect the cerebral cortex (parietal lobe involved in spatial orientation and frontal lobe involved in cognition), basal ganglia (especially substantia nigra, whose neurons secrete dopamine involved in the control of movement and posture), cerebellum (involved in movement and balance control), vestibular (involved in head movements' detection), visual (involved in orientation in space), and the ankle capsulo-ligamental (involved in ankle sensitivity and stabilization) systems and disturb postural control [2–9]. Pathological postural attitudes such as idiopathic scoliosis also affect postural control [10]. Given the multitude of structures involved in postural control and because of its complexity, many other pathologies are likely to disturb postural control.

Although pathologies can alter postural control in a nonspecific way, for a given pathology known for affecting particularly the postural function, the postural behavior evolves specifically (e.g., [11, 12]). For caregivers, postural control tests can thus help to determine the pathology in question (or the diagnosis) in patients (e.g., [13–15]). However, it is essential to use adequate evaluation methods and techniques which give reliable quantitative and qualitative variables in order to pinpoint the functional state of the sensory, central, and motor components of the postural function.

Nevertheless, as regards the literature, one can observe that the different techniques and methods employed, as well as the different quantitative and qualitative variables measured, in order to objectify postural control, are often chosen either arbitrarily or on the basis of materials classically used by the authors without taking into account the population under consideration, the objective of the postural task, and the environmental conditions. For these reasons, the aim of this review was to present and justify the different testing techniques and methods with their different quantitative and qualitative variables to make it possible to precisely evaluate each sensory, central, and motor component of the postural function in pathological subjects but also in healthy subjects since they constitute the benchmark in terms of postural behaviour.

## 2. Principles of Analysis of Postural Control

### 2.1. Quantitative and Qualitative Analyses

Postural control can be quantitatively considered by measuring the movement of the centre of mass (COM), the centre of foot pressure (COP), and body segments but also by measuring electromyographic activities and evaluations of the contribution of different sensory information. The qualitative analysis consists of describing how postural control is organized in relation to the mechanical and neurophysiological aspects.

### 2.2. Postural Performance

Postural control can be characterized in terms of performance according to the postural condition under consideration. Postural performance refers to the ability to maintain body balance in challenging postural conditions (e.g., a stance classed as a handstand, monopedal dynamic stance) and thus avoiding postural imbalance and falls. Postural performance can also characterize the ability to minimize body sway in more conventional postural conditions (e.g., bipedal quiet stance).

### 2.3. Postural Strategy

It can be defined on the basis of the spatial and temporal organization of different body segments as well as the extent and order of recruitment of different muscles activated. The different sensory sensors involved in postural regulation as well as the weight of different sensory information and/or the preferential involvement of different neuronal loops can also contribute to describe postural strategy.

## 3. Testing for Postural Performance and Strategy

### 3.1. Testing for Postural Performance

The ability to ensure postural stability in challenging postural conditions can be evaluated with practical or experimental tests with different postural stances (e.g., bipedal stance, monopedal stance) on small bases of support and moving platforms leading to expected and unexpected postural disturbances. Subjects retain their body balance or not and then pass the test on offer or not which corresponds to a certain performance level. If the test consists of discriminating between the ability to minimize body sway in easy and unspecific postural conditions, different instrumented evaluation methods can be employed.

### 3.2. Testing for Postural Strategy

The use of instrumented evaluation methods is sometimes insufficient to precisely characterize the postural strategy employed by subjects. Evaluation of the contribution of each component of the postural function often involves motor disturbance (mechanical disturbance), sensory stimulation (sensory manipulation), or cognitive disturbance (e.g., virtual simulation, dual-task) protocols. Methods combining these different techniques also provide relevant information in analysis of the postural function.

## 4. Basic Noninstrumented Postural Tests

Most of the time, the evaluation of postural function requires technological materials but simple tests can also be used to identify postural dysfunctions in aged and frail subjects and subjects with pathologies (acute and chronic pathologies). However, basic postural tests were mainly designed to evaluate older subjects' postural abilities as well as their risk of falling whereas there are only a few tests for subjects with pathologies. A number of tests exist, such as the Berg Balance Scale [16, 17], Timed Up-and-Go [18, 19], Tinetti test [20], Short Physical Performance Battery [21], Mini Balance Evaluation Systems Test [22], Unified Balance Scale [23], Functional Ambulation Classification [24, 25], and the Postural Assessment Scale for Stroke patients [26] for example. Currently, the Berg Balance Scale or Mini Balance Evaluation Systems Test would be the tests particularly recommended by certain experts [27]. Moreover, it is known that failure to maintain the monopodal stance for 5 seconds constitutes a strong risk of falling for older people even if this very discriminating test on its own does not predict all falls that might occur in their life [28]. This monopedal stance can be suggested for 30 seconds (3 trials), and either the subject passes the test or he/she fails. In the event of the latter, one can record the holding time from the best trial (if this is less than 14 seconds, postural abilities are considered very weak). Moreover, walking speed tests over a 4-metre distance with a chronometer also make it possible to evaluate the postural abilities of older subjects. For example, a walking speed corresponding to 0.8 m·s^−1^ is predictive of weak functional abilities while a speed corresponding to 0.6 m·s^−1^ constitutes a threshold below which the risk of falling is critical [29]. However, these practical tests are of interest to subjects whose postural abilities are very weak but they do not make it possible to carry out qualitative analyses of postural control, especially for (young) subjects with pathologies. Only technology and instrumented tests offer this possibility.

## 5. Material and Technology for Instrumented Tests

Even though noninstrument tests can be useful to the clinician in diagnosing sensory-motor disorders, they only provide a gross indicator of postural control efficiency. Detailed analysis of postural control performance and associated strategies require the use of instrumented tests with various materials to make it possible to carry out kinetic, kinematic, and electrophysiological analysis.

### 5.1. Kinetic Devices

Force platforms are the most widely used devices in assessing postural function. Force platforms are made of a dimensionally stable board under which load sensors are positioned. They can be incorporated in specific motorized or nonmotorized devices in order to generate instability.

#### 5.1.1. Main Technologies

The most widely used nonmotorized devices are wobble boards, usually made of wood or plastic materials, with hemispherical or hemicylindrical bases (seesaws) that create instability in all spatial directions or a given plane [30]. Instability can be modulated according to the radius and height of the base. While reducing ground surface contact and raising feet surface contact, wobble boards challenge both sensory and motor components of the postural control system [31]. Indeed, standing on a wobble board requires the centre of mass (COM) to be projected onto the board's point of contact with the floor [32], thereby increasing postural sway and challenging the postural control system when compared to standing on stable ground [30]. Wobble boards sometimes include autonomous measurement devices—mainly potentiometers recording the discrepancies of the seesaw from the horizontal plane—and do not require the use of a force platform [33]. Although such devices are affordable and can be used for sports training and balance rehabilitation, they only provide a macroscopic postural sway index without directional characteristics that are required for a suitable assessment of postural function. Since the pioneering work by Nashner [34], many studies have been conducted with servo-controlled motorized force platforms. Most current advanced devices can provoke cyclic or sudden translational movements in the medial lateral (ML) and/or anterior/posterior (AP) direction and rotational movements in all directions or a given plane.

When focusing on the technology of force platforms, two “families” of platforms can be considered: (1) those equipped with monoaxial load cells that only measure the vertical component of the ground reaction force (FZ), usually with at least three strain gauges (uniaxial plates) and (2) those equipped with load cells (usually four strain gauges or piezoelectric sensors) that measure the three components of the ground reaction force (FX, FY, and FZ) and the moment of force acting on the plate (MX, MY, and MZ) (multiaxial pates) [35]. Both uni- and multiaxial plates can be used to calculate the ML and AP time series of the centre of pressure (COP, the point of application of the vertical ground reaction force) over time during a postural test. The COP is the most measured parameter to assess postural function. Postural sway is commonly applied to variations in the COP position, whereas displacements of the COM are applied to body sway [36]. With multiaxial plates, the relative horizontal COM displacements can be calculated thanks to a double integration of the ML and AP components of the ground reaction force (divided by the mass). Nevertheless, it is not possible to calculate the initial velocity and position of the COM, even though some methods have been suggested to estimate these initial constants [35]. COM horizontal positions can also be evaluated from COP displacements measured with both uni- and multiaxial platforms by using an inverted pendulum model and a filtering method based on the COM/COP relationship in the frequency domain [37]. Nevertheless, only kinematic analyses make calculation of COM motions in three spatial directions possible.

Force platforms initially designed to be used as video game controllers have also been recently suggested as very affordable tools to assess postural function. Many studies have been conducted in order to compare multiaxial platforms with these particular unidirectional platforms, characterized by inconsistent and low sample rates with a large amount of irrelevant results. Such devices tend to overestimate COP parameters such as velocity [38]. The overestimation of COP parameters appears to be a typical feature of uniaxial force plates and depends on the postural task's complexity—the easier the postural task, the larger the overestimate [38, 39]. Despite this limitation, monoaxial force plates provide appropriate accuracy for most standing balance assessments. Nevertheless, measurements from unidirectional and multiaxial platforms should not be used interchangeably [39]. Whatever the type of platform used, they must meet further requirements whose standards have been recently updated [40]: accuracy should be better than 0.1 mm, precision should be better than 0.05 mm, resolution should be higher than 0.05 mm, frequency bandwidth should be 0.01–10 Hz, and linearity should be better than 90% across the whole range of measurement parameters.

Since the COP comes from the muscle actions of both feet, the use of two platforms placed side by side can be required in order to analyze in detail the balance mechanisms in the frontal plane by measuring the ground reaction forces under each foot [41, 42], especially if body weight distribution asymmetry is suspected, as with hemiparetic or amputee patients [43]. It is also possible to distinguish the hip loading/unloading mechanism from the ankle inversion/eversion mechanism acting on the frontal plane with two platforms [42]. Some force plates also make it possible to separately analyze the COP movements at the heel and those of the metatarsus under each foot. Some authors have developed specific measurement devices to analyze more complex postural conditions. Examples include the concomitant use of the force platform and force transducers positioned on handles to analyze postural tasks performed while using hand supports [44] and specific ergometers equipped with 3D load sensors positioned on feet and hand supports to analyze horizontal [45] and vertical [46] quadrupedal postures. Devices using pressure sensors as flexible instrumented insoles [47] or pressure plates [48] can be used to measure plantar pressure distribution, especially when modulating conditions related to footwear. Plantar pressure measurements provide information regarding potential impairments of the foot and its disorders [49]. Among all these kinetic devices, force platforms are considered to be the gold standard [38], with COP being the most widely measured parameter from which various variables can be calculated to assess postural function [35].

#### 5.1.2. Main COP Variables

Raw COP recordings are mainly used by clinicians and researchers as gross visual representations of the output of the postural control system. Two representations can be obtained, the statokinesigram (construction of the COP map in the horizontal plane) and the stabilogram (time series showing variation of the COP in the AP and ML directions). Nevertheless, the calculation of other COP variables from raw COP data is necessary in order to analyze the mechanisms involved in postural regulation. COP variables can be categorized as global and structural variables [35, 50–52]. Global variables characterize the magnitude of the resultant and/or the ML and AP components of the COP traces in both time and frequency domains. Authors usually consider that the greater the magnitude or deviations of a global variable, the poorer the postural stability. Nevertheless, global variables are not sensitive to the structure of variation which can potentially provide essential insights into the postural control process in a variety of contexts [50, 51]. Hence structural variables can be considered. These variables decompose the COP sway patterns into subunities and correlate them with the motor control process [35, 50–53].

*(1) Global COP Variables*. Many different global variables have been put forward [50]. Making an exhaustive list of all these variables is not the concern of this study and only the most common and relevant ones are given here and commented on.

- Mean coordinates reflect the topographical features of plantar pressure distribution [54] and depend on the position of the subjects on the force plate. They can be influenced by wearing specific shoes (e.g., ski-boots [55]) and anthropometric characteristics [56]. They can also be used as a clinical index to detect specific pathologies resulting from bilateral unbalance.
- Ellipse area/surface quantifies 90 or 95% of the total area covered in the ML and AP direction using an ellipse to fit the data. It is considered to be an index of overall postural performance—the smaller the surface, the better the performance [57]. Caution must be taken when calculating this variable and the use of prediction ellipses should be preferred to confidence ellipses [58, 59].
- Path length quantifies the magnitude of the two-dimensional displacement based on the total distance travelled. It is considered to be a valid outcome measurement in numerous populations and balance conditions—the smaller the path length, the better the postural stability [39].
- Amplitude of displacement is the distance between the maximum and minimum COP displacement for each direction—the greater the values, the worse the postural stability. COP amplitude is a reliable parameter which has been widely used in order to analyze postural deficits with patients suffering from neuromotor disorders such as cerebral palsy, especially when analysis was conducted on the ML direction [60].
- Velocity is calculated by dividing the COP excursion by the trial time. One can consider the ML and AP components or the resultant velocity. It reflects the efficiency of the postural control system (the smaller the velocity, the better the postural control) while characterizing the net neuromuscular activity required to maintain balance [61] and has been considered as the measurement with the greatest reliability among trials [35]. Additional authors considered COP velocity as the most sensitive parameter in comparing individuals from different age groups and with different neurological diseases [62–64]. Vsetecková and Drey [65] also underlined the major role of COP velocity in the feedforward mechanisms of the postural control system during quiet stance.
- Standard deviation (SD), root mean square (RMS): RMS is defined as the square root of the mean of the squares of a sample. If the COP signal has zero mean, RMS and SD provide the same result. RMS and SD are variability indexes of COP movements which offer good reliability in discriminating between young and older subjects and subjects who are healthy and those with pathologies [35, 66–68].
- Total power frequency is considered an energy-expenditure index.
- Mean, median, centroid, and 80–95% power frequency: these parameters provide a general view of the frequency content of the COP signal. Higher frequencies of postural sway are indicative of postural control with faster and smaller postural adjustments [69]. Mean and median frequency can also be viewed as indexes of ankle stiffness—the higher the frequency of postural sway, the higher the stiffness around the ankle joint [70]. 80–95% power frequency characterizes the frequency band with 80–95% of the spectral power. Baratto et al. [50] suggest 80% of spectral power is the best value to characterize modifications in the postural control system.
- Frequency bands distribution: the frequency content of the COP signal is studied by incorporating amplitudes within frequency bands in order to characterize the preferential involvement of specific neuronal loops in postural regulation [71–74]. Three frequency bands are usually considered: low frequencies (0–0.02/0.5 Hz) which mostly account for visuovestibular regulation, medium frequencies (0.2/0.5–2 Hz) for cerebellar participation, and high frequencies for proprioceptive participation (>2 Hz), the limits of these bands being different according to the authors [66, 71].

Spectral analyses of COP sway are usually performed with algorithms based on Fourier transforms [35, 69]. These methods should be used with caution since the COP can demonstrate nonstationary characteristics [69, 75]. Computational approaches such as discrete wavelet analysis [75] or empirical mode decomposition [69] are more suitable for nonstationary signals.

*(2) Structural COP Variables*. Because of the nonstationary characteristic of the COP signal, standard time and frequency analysis methods cannot adequately describe the dynamic changes of postural sway. Because the postural control system must be considered as a nonlinear system (where reactions are not proportional to the applied stimuli), various methods of nonlinear dynamics and quantitative descriptors have been put forward for the analysis of the COP signal [76].

Collins and De Luca [53] introduced a method for analyzing time evolutionary properties of the COP known as stabilogram diffusion analysis. They assumed that maintenance of erect posture could be considered as a stochastic process governed by the laws of probability. Stochastic analysis is focused on the evolution of complex structures resulting from interactions between numerous elements. From a stochastic perspective, the COP time series is considered as the performance of a theoretical process consisting of random variables relating to points in time, with this random theoretical process being analyzed by performing a statistical inference on its properties [77]. Collins and De Luca [53] decomposed the COP signal into two stochastic processes modelled as Brownian fractional movements: a long-term process with a large exponent characterizing a persistent structure and a short-term one with a small exponent characterizing an antipersistent structure. These two structural sub unities were considered to, respectively, characterize the closed and open-loop mechanisms of human postural control [53]. Nevertheless, the existence of a critical point in time that distinguishes open- and closed-loop processes in postural control has been challenged and the functional significance of this model is no longer widely accepted [78, 79].

With the rambling-trembling hypothesis, Zatsiorsky and Duarte [80] put forward an alternative method which also differentiates between two timescale components in the COP signal. In the context of the equilibrium-point hypothesis [81], they suggest that equilibrium is adopted according to a migrating reference point, characterized by the conservative rambling subsystem, whose movements reflect an exploratory behaviour which does not induce substantial restoring forces. The oscillations around this reference point characterize the operative trembling subsystem which aims at maintaining equilibrium around the reference point thanks to restoring forces [82]. Rambling and trembling subsystems describe two different processes in the control of an upright stance: rambling reflects the supraspinal processes involved in the control of the movements of the reference point, whereas trembling reflects spinal reflexes and changes in the intrinsic mechanical properties of the muscles and joints [82].

Another approach of COP structural analysis is based on the assumption that the postural control system is a chaotic system with a deterministic nature [76]. Fractal dimension methods have been put forward in order to detect chaos in posturographic signals. Decreased postural stability due to lack of visual cues [76] or neurological pathologies [83] is characterized by an increase in the signal's fractal dimension. Fractal analysis of COP signals represents a reliable and sensitive tool to assess subtle changes in postural control caused by a pathology and/or age [76, 83]. Sample entropy (SampEn, a measure of regularity), approximate entropy (ApEn, a measure of unpredictability), and Lyapunov exponent (LyE, a measure of divergence) are nonlinear dynamic parameters that can be extracted from COP plot points in order to perform structural analyses [52, 84, 85]. Significant regularity in postural control resulting in low values for SampEN, ApEn and LyE characterizes constraint systems with reduced adaptation and response aptitudes to potential disturbances and increased risk of falling. Patients suffering from neurological disorders typically demonstrate lower SampEN, ApEn, and LyE values compared to healthy subjects and this reflects impairment in postural function [85, 86]. Unconstrained and irregular postural oscillations reflect the efficiency of postural control related to the complex mechanisms with structured variability but not exact repetition [87].

Additional authors have put forward other specific methods for COP structural analysis. One can mention the sway-density curve concept from Baratto et al. [50], based on the idea that COP movements are incompatible with Brownian movement, the structural analysis proposed by Duarte and Zatsiorsky [88], which requires carrying out prolonged postural tasks in order to identify timescale components in the COP signal, the empirical mode decomposition put forward by Pachori et al. [69], which decomposes the COP signal into intrinsic mode functions (i.e., local oscillations that compose the raw COP signal), the entropic half-life approach from Baltich et al. [89], which makes it possible to quantify the SampEn of the COP with different time scales without affecting the signal length, or the rotary spectra approach proposed by Agostini et al. [90], which separates rotational iso-frequential components of the COP signals from nonrotational ones. These approaches are developments of preexisting methods detailed more precisely above or which require more research in order to test their reliability and validate their relevance for clinical applications.

### 5.2. Kinematic Devices

#### 5.2.1. Main Technologies

Even though basic video recordings can provide both qualitative and quantitative information about segmental postural strategies, especially when using specific advanced software [91], only 3D motion capture systems offer the high level of accuracy and reliability necessary to record the small motions which characterize the unperturbed upright stance [92]. Two different technologies can be identified. Passive marker systems use reflective markers with a set of further high-resolution, high-speed cameras with incorporated infrared/near infrared strobes. The cameras record the reflection from the markers placed on specific anatomic landmarks whose identification is performed thanks to the software. Active marker systems use powered markers sending an infrared signal which is captured by sensor units. Each active marker has its proper frequency. Active marker systems avoid the postprocessing identification procedures required with passive marker systems but require small powered boxes to be attached to the subjects' skin.

The use of 3D body-worn accelerometers has recently been suggested as an alternative to force platforms for measuring postural sway [93]. Accelerometers can be positioned on the posterior trunk to give an estimate of COM movements [93] or on specific joints to assess joint movements and/or COM movements thanks to subsequent modelling and calculation [94]. Accelerometer-based devices provide a sensitive means of measuring subtle balance deficits in clinical settings [93, 94].

Electrogoniometers make it possible to measure joint angular displacements and have been mainly used to analyze changes in segmental postural coordination while using the dynamic approach to postural control (e.g., [95]). Electrogoniometers provide a first level of accuracy, which is acceptable for dynamic postural tasks [95], but it might be inadequate for measuring joint movements in static postural tasks with healthy subjects.

Laser-displacement sensors can also offer interesting possibilities for kinematic measurements in order to compute joint angle measurements [96, 97] or to analyze the movements of a specific body landmark like a lumbar vertebra, whose movements can be incorporated into a procedure to estimate COM displacements [64]. Laser displacement sensors can provide a very high level of accuracy, making it possible to get reliable measurements of angular motion for subsequent derivative calculations [96].

#### 5.2.2. Main Kinematic Variables

Because of the complexity of the musculoskeletal system, kinematic analyses are always associated with using a biomechanical model with a different degree of complexity. Biomechanical models usually consider the body as a system made up of rigid articulated segments—the more segments and the more freedom of the joints, the more complex the model. Whatever the complexity of the model, the calculation of joint angles can be viewed as a first step that makes it possible to characterize skeletal alignment and assess the overall segmental postural organization [98]. Velocity, acceleration, and jerk calculations provide additional information about joint movement characteristics. Joint moments can be calculated by inverse dynamics when performing more complex analysis combining force plate and kinematic measurements [92]. While using accelerometric devices on the belt to quantify postural sway, Mancini et al. [93] have shown that jerk was the most discriminating parameter to differentiate sway in subjects with Parkinson's disease compared to healthy control subjects. It must be noted that classic movement descriptors can be calculated independently of the type of kinematic device employed while using integration/derivation procedures with accurate filtering and data smoothing procedures [41].

As COM is the only variable that characterizes body sway, its calculation has been of major importance particularly in understanding the relationships between the COM and COP [36]. Despite the widespread use of COM, its calculation is a complex and time-consuming operation which requires a multisegmental model of the body. Winter et al. [99] recommends a 14-segment model with 21 markers. Segmental inertial characteristics must also be estimated thanks to anthropometric tables [99] or optimization procedures [100]. Once the COM is being calculated, similar parameters to those described in Section 5.1.2 with the COP can be calculated.

The analysis of interjoint coordination is a major concern when studying multisegment movement strategies of postural control. Many methods have been put forward in order to identify joint synergies and/or quantify the respective contributions made by joint motions in the control of COM or COP, such as principal component analyses [101], multivariate canonical correlation analyses [102], coherence and cophase analyses [103], coherence spectrum analyses [96], relative phase estimates [104], cross-correlation analyses [92], or wavelet coherence analyses [105]. Similar analyses can also be conducted in order to analyze organization and coordination of physiological tremors during postural tasks [106].

### 5.3. Electromyography

Electromyographic (EMG) recordings have been widely used in the assessment of postural function. Amplitude, temporal, and frequency parameters can be differentiated [107]. Temporal EMG analyses have been extensively used in order to characterize postural responses following platform-movement disturbances or anticipatory postural adjustments with voluntary movements [108] when identifying bursts of muscle activity [109]. EMG amplitude analyses, like RMS or area calculations [55], are used to reflect the magnitude of muscular activity in maintaining specific postural tasks. Frequency domain analyses have been used with moving oscillating platforms and have shown that increased frequency of platform oscillations increases the amplitude spectrum of muscle activity [110].

EMG recordings can also be used in order to study postural segmental strategies and interjoint coordination. Cross-correlation analysis can be applied to investigate the relationships between COP/COM time series and time domain EMG data [111]. The EMG activity of postural muscles during stable standing tasks has also been analyzed in the frequency domain with coherence analysis in order to determine coordination patterns [112].

## 6. Main Postural Conditions

### 6.1. Postural Stances

**Figure 1:**
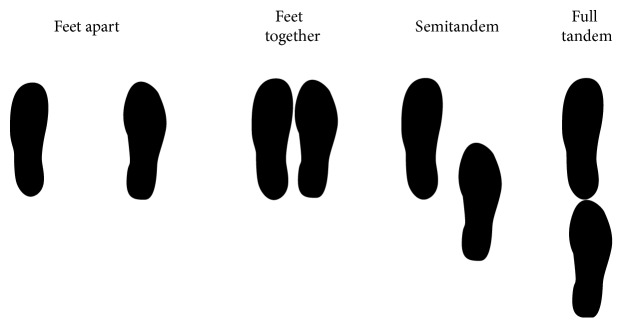
Illustration of the different foot positions.

Generally speaking, postural tests are done either in a bipedal stance or in a monopedal stance. In a bipedal stance, the feet are positioned according to a multitude of possible conditions, the main ones being: feet apart, feet together, semitandem, and full tandem (Figure 1). The legs are generally straight (they can be also flexed if required for the protocol) and the feet either form an angle of 15–30° or are positioned parallel (e.g., [113]). The intermalleolar distance is usually 5 cm for an angle of 30° and 10–15 cm for an angle of 15° [61, 113] and varies between 0 and 15–20 cm for quasi-parallel feet [114]. In a monopedal stance, the supporting leg can be the dominant leg or the nondominant leg according to the aim of the postural test (the dominant leg is the one used for kicking a ball). The supporting leg can be extended or flexed according to the requirements of the experiment. The nonsupporting leg can be either raised and flexed 90° at knee-level or lifted so that the subject's big toe touches the medial malleolus of the supporting leg lightly (no support). The two hips are placed in a neutral position (0° of flexion or slight flexion if the toes touch the medial malleolus) if the supporting leg is extended, which is different if the supporting leg is flexed. Whatever the postural stance chosen from among the different possibilities mentioned above, subjects stand in a relaxed manner with arms extended out to the sides or crossed in front of their chest. When arms are moving freely, postural performance is modified [115].

In certain circumstances with pathological subjects or healthy highly skilled subjects, other postural stances can be adopted. Hence, the other possible main supports are ischium (seated with or without feet support), knee (kneeling), and hands (a stance classed as a handstand for skilled sportsmen or quadrupedal postures) [45, 46, 57].

For all the postural stances mentioned above, other body segments can be also used as additional supports: hand (one or two), trunk, head, thigh, shank, arm, and forearm. Analyses of the contribution of haptic information (proprioception and cutaneous sensitivity combined) in postural regulation as well as the application of different biomechanical conditions justify the use of these additional supports.

### 6.2. Visual Condition

Most of time the postural tests are completed without and/or with visual information. The suppression of visual cues may occur through closing the eyes or blindfolding but also by putting subjects in total darkness. Moreover, the contribution of visual cues (calculated with quantitative and qualitative variables obtained in the closed eyes condition compared to the same variables measured in the open eyes condition) constitutes relevant data in the analysis of postural control in subjects who are healthy and who have pathologies.

### 6.3. Balance Condition

Both static and dynamic conditions are used when testing postural control. For subjects with pathologies, it is prudent to start with postural tests in static conditions. Dynamic conditions are more discriminating than static conditions in terms of postural control [5]. The contribution of visual cues is essential in static conditions while the contribution of proprioception inputs is fundamental in dynamic conditions [56]. However, when the difficulty of postural task increases in dynamic conditions, the contribution of visual information increases [5].

### 6.4. Duration of Tests

In the literature, one can find different durations of test for evaluating postural control. Generally speaking, the duration of tests in static conditions is longer than that observed in dynamic conditions. One can estimate that appropriate durations mainly vary between 20 and 60 s for static conditions and between 10 and 30 s for the dynamic conditions depending on the difficulty of the postural task and the population under consideration (e.g., subjects with pathologies, older subjects, highly skilled subjects). In static conditions, a 20-s duration would be the minimum under which the postural test may lose consistency since the stationary process (stationariness of the posturographic signals) of postural control requires some seconds of adjustment time [40, 116]. The last meeting of the International Society for Posture and Gait Research suggested that from a recording time of 25–40 s the posturographic parameters are steady and reliable and a reasonable comprise could be 30 s with 5 s of adjustment time before starting the recording [40]. In turn, the complexity of evaluation protocols sometimes involves longer durations in specific physiological and/or psychological (or cognitive) conditions. Nevertheless, the experimenter should ensure that the test duration does not cause fatigue especially in subjects with pathologies. In dynamic conditions, a 30-s duration seems to be the maximum in order to avoid fatigue in healthy subjects, while this duration should be shorter for subjects with pathologies. A 15/25-s duration for healthy subjects and a 10/20-s duration for subjects with pathologies seem to be appropriate.

## 7. Disturbing Postural Conditions

Different evaluation methods make it possible to explore each component of the postural function with motor disturbance (mechanical disturbance), sensory stimulation (sensory manipulation), and/or cognitive disturbance (e.g., cognitive task associated to postural balance maintenance) protocols.

### 7.1. External Mechanical Disturbance

The first principle making it possible to destabilize body balance consists of mechanically creating COM displacements thanks to external disturbances. To this end, unexpected disturbances produced by percussion or pushing a large body segment such as the trunk can produce mechanical disturbances which require effective postural reactions in order to maintain body balance. The second principle consists of modifying the state of the base of support with moving platforms (e.g., translation, pitching, rolling or yaw movements) and surface reductions to this base. Finally, the third principle consists of applying articular constraints by limiting or blocking joint movements (cervical and lumbar spine, hip, knee, and ankle) by means of orthotic devices, specific equipment (specific shoes or clothing), collars, and so forth. This principle involves mechanical compensation of joint constraints by changes in postural strategy by reorganising muscle coordination which is made possible by inherent redundancies in the human body [55]. These particular constraints result in not only mechanical constraints but also sensory disturbances since a mere cervical collar effectively joins the head and trunk which limits the information from cervical articulations [117].

### 7.2. Sensory Disturbance

**Figure 2:**
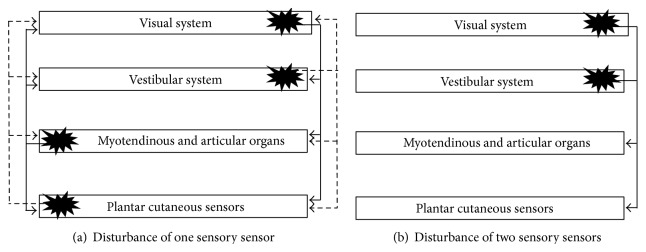
The disturbance of one (a) or two (b) sensors leads to an increase in the sensory contribution of other sensors (not disturbed) in postural regulation. The disturbance is indicated by a star-shaped sign while the increase in sensory contribution is indicated by an arrow.

In order to study the contribution of different sensory sensors to postural regulation, the experimenter often uses sensory manipulations of one (simple manipulation) or two/three (combined manipulation) sensory sensors. The disturbance in one or several sensory sensors impacts the contribution of other sensory sensors (Figure 2). The sensory manipulation technique makes it possible to evaluate the efficiency of different sensory sensors (i.e., the ones that are not manipulated and make it possible to regulate postural control), to identify the predominance of a particular sensor among all the sensors or the preferential use of certain sensory information (i.e., the sensor that when manipulated induces greater postural disturbances than when the other sensors are individually manipulated), and to define the capacities to compensate and/or switch the different sensory inputs (i.e., the abilities to limit the effects of postural disturbance through the increased contribution of sensory sensors which have not been not manipulated) [118–120].

#### 7.2.1. Visual Disturbance

The alteration of visual cues can be generated through the reduction or suppression of brightness and/or field visual. The experimenter can reduce the visual flow with stroboscopic light, light filters and other processes intended to limit the availability of visual information. He/she can also move the visual target away from the subject in order to attenuate the visual effects on postural control [121]. Visual disturbances can also be created by giving erroneous visual cues through the application of the optokinetic technique [121]. This makes it possible to project a moving visual scene on a subject/patient who is standing. It triggers nystagmus in the direction selected by the experimenter and causes postural deviation [122]. An optokinetic stimulus induced by the rotation of a disc from the left side to the right side causes an inclination of the body to the right side to compensate for the body motion illusion to the left. The purpose is not to destabilize the subject/patient, but to provoke neurosensory conflicts since proprioceptive, vestibular, and plantar cutaneous inputs indicate no movement.

#### 7.2.2. Vestibular Disturbance

In order to study the contribution of vestibular inputs in postural regulation, the vestibular afferences can be disturbed with particular electrical stimulations. These disturbance stimulations are done with the galvanic vestibular stimulation technique. It consists of provoking neurosensory conflicts by applying an electrical current via surface electrodes to the mastoid processes [118, 119, 123]. This electrical current disturbs the transduction of ciliated cells in ampullary crests (in semicircular canals) and macula (in otoliths) which induces body motion illusions [123, 124] and modifies postural attitude [125–127] but does not change the internal representation of the subjective vertical [127]. Galvanic vestibular stimulation can be applied unilaterally or bilaterally through monopolar or bipolar stimulus [128, 129]. A bilateral and bipolar stimulation provokes tilting on the medio-lateral axis towards the anode electrode [130]. Bilateral and monopolar stimulation creates tilting on the anteroposterior axis, backward for anode electrodes, forward for cathode electrodes [129]. The head should be vertically placed (not inclined) because its position can influence the postural response [131]. The intensity of stimulation influences the postural response—the higher the intensity, the greater the postural reaction [129, 130]. The disturbance intensities raised in the literature go from 1 mA to 5 mA [124, 132]. Higher intensities are feasible but would not be harmless in terms of the risk of burning the subject's skin [132]. The delay in postural response to stimulation is about 1-2 s [124]. The experience of more natural stimulation of the vestibular system, that is, through accelerations of the head movement through specific physical activities (e.g., control subjects versus pilots), can limit the magnitude of body deviation [125]. This study showed that pilots have a stronger ability to suppress vestibular illusions than control subjects. Moreover, the risk of body destabilization (falling) of subjects/patients is real when using galvanic vestibular stimulation, so the experimenter must ensure he/she applies progressive intensities especially with subjects who are impaired or have pathologies. For example, individuals with Down's syndrome showed greater sensitivity to galvanic vestibular stimulation than control subjects and were not able to select the appropriate motor strategy to efficiently maintain balance and compensate for the effects of galvanic vestibular stimulation [133].

#### 7.2.3. Proprioception Disturbance

The proprioceptive disturbance is mainly studied by manipulating myotatic and tendon sensors since the manipulation of articular sensors is done with articular constraints or blocking. Tendon vibration and neuromuscular electrical stimulation are the two techniques mainly used to disturb the myotendinous complex.

Tendon vibration applied onto muscle belly or tendon modulates the afferences of fibres of type Ia [134, 135]. Muscle spindle secondary endings (fibres II) and Golgi tendon organs (fibres Ib) would be either insensitive or only slightly sensitive to tendon vibration in relaxed muscles [135]. Tendon vibration induces perceived muscle stretching as well as body motion illusion which results in modification of body orientation [136]. Vibratory stimulation provokes body inclination backwards when it is applied to the triceps surae [137] and provokes body inclination forwards when it is applied to the tibilis anterior [138]. The vibratory frequency and amplitude usually used are, respectively, between 30 and 100 Hz and between 0.2 and 3 mm [135, 139–141]. The stimulation frequency influences the muscle response—the higher the values, the greater the postural reaction. Vibrations below 20 Hz induce mechanical resonance [142]. Finally, the risk of body destabilization in subjects/patients is real when using tendon vibration, so the experimenter must ensure he/she applies progressive frequencies especially in subjects who are impaired or who have pathologies.

Neuromuscular electrical stimulation can also be employed to disturb the contribution of myotatic loop in postural regulation [117, 143]. It is applied either onto muscle belly or on nerve. The frequency and intensity values of stimulation probably influence the disturbance effects on postural control but they are currently unknown.

#### 7.2.4. Plantar Cutaneous Disturbance

Overall, there could be three main techniques to reduce or suppress plantar cutaneous sensitiveness. The first technique consists of anesthetizing the sensitivity of cutaneous receptors through hypothermia by placing the plantar sole in iced water (0–2°C or 0–5°C) for some minutes (e.g., 10 or 20 min) in order to disturb postural control [117, 139, 144]. The second technique consists of using a foam-supporting surface which appears to be an appropriate tool to challenge postural control and produces substantial and multidirectional balance disturbance [145, 146]. Static standing on a foam surface would change the multiple biomechanical variables in the foot, resulting in an alteration to the distribution of plantar pressures [147]. The third technique consists of provoking ischemia by partially blocking blood circulation in the ankle or thigh [148–150]. Ischemia produces local metabolic changes that would alter the sensory pathways and would consequently affect the activity of the muscles involved in postural control [151]. This study suggested that these changes would cause a decrease in the monosynaptic facilitation of homonymous motoneurons linked to afferents Ia and a polysynaptic disfacilitation in motoneurons linked to cutaneous afferents. In a clinical context, the foam-supporting surface seems easier to safely use than the cooling technique (hypothermia) and especially the ischemia technique in order to study the contribution of plantar cutaneous inputs in postural regulation.

#### 7.2.5. Combined Materials

This type of device comprises a force platform and a cabin which can be mobilized (tilted) either together or separately. Tilting the platform and/or the cabin combined with the elimination of visual information consists of creating sensory conflicts. Tests are performed in different sensory conditions in order to study how subjects cope with modifications to the environment. This type of device makes it possible to conduct postural evaluations in different sensory conditions:

- all the sensory information is available,
- the visual information is eliminated: blindfolded,
- the visual information is disturbed: the cabin is tilted (eyes open),
- the proprioceptive information is modified: the force platform is tilted,
- the visual information is removed and proprioceptive information is changed, blindfolded, and the cabin is tilted,
- the visual and proprioceptive information is inadequate: the platform and the cabin are tilted.

### 7.3. Cognitive Disturbance

Postural control system is not totally autonomous and requires attentional resources [152]. Many studies have produced evidence that the attentional demands of postural control increased with ageing, the difficulty of the postural task, the absence of information from a sensory system, and pathology or injury [153–157]. The investigation of the attentional demands of postural control broadly involves the use of dual-task paradigms [152, 157]. Dual-task paradigms are based on the assumption that the central nervous system has limited processing resources and when two tasks are performed at the same time, they can interfere if they imply the use of shared resource requirements from similar specialized structures [152, 158]. Hence, when postural control is associated with a secondary cognitive task, interference implies a shared requirement for attentional processes. Dual-task paradigms can be used to focus on just the attentional demands required for postural control during a cognitive task [159, 160]. Cognitive tasks such as a calculation task, memory task, visual search task, or verbal fluency task, as well as tasks based on biofeedback techniques (e.g., games-based balance exercise), are generally undertaken simultaneously during postural tasks [161]. Other tasks such as the ones which imply responding as quickly as possible to auditory, visual, and sensory signals make it possible to evaluate reaction time alone or combined with postural tasks. According to the population under consideration (e.g., subjects with pathologies and who are older and subjects who are healthy and sportsmen), cognitive tasks can be developed to suit their abilities.

## 8. Conclusion

Currently, whatever the population under consideration (healthy or subjects with pathologies), the objective of the postural task and the environmental conditions, postural control can be appropriately evaluated in terms of postural performance and strategy by using reliable technological tools and tests. However, all the theoretical considerations related to the postural function are not yet experimentally verifiable through postural analyses. Refinement in the analysis of the contribution of sensory, central, and motor components to postural behaviour is subject to future technological progress as well as advances in knowledge about postural function.
